# Classifying Comments on Social Media Related to Living Kidney Donation: Machine Learning Training and Validation Study

**DOI:** 10.2196/37884

**Published:** 2022-11-08

**Authors:** Mohsen Asghari, Joshua Nielsen, Monica Gentili, Naoru Koizumi, Adel Elmaghraby

**Affiliations:** 1 Department of Computer Science and Engineering University of Louisville Louisville, KY United States; 2 Department of Industrial Engineering University of Louisville Louisville, KY United States; 3 Schar School of Policy and Government George Mason University Washington, DC United States

**Keywords:** living kidney donation, kidney donation, kidney transplantation, text mining, web scraping, NLP, deep learning, neural network, barriers to kidney donation, barriers, awareness, perception, machine learning, online source, online comments

## Abstract

**Background:**

Living kidney donation currently constitutes approximately a quarter of all kidney donations. There exist barriers that preclude prospective donors from donating, such as medical ineligibility and costs associated with donation. A better understanding of perceptions of and barriers to living donation could facilitate the development of effective policies, education opportunities, and outreach strategies and may lead to an increased number of living kidney donations. Prior research focused predominantly on perceptions and barriers among a small subset of individuals who had prior exposure to the donation process. The viewpoints of the general public have rarely been represented in prior research.

**Objective:**

The current study designed a web-scraping method and machine learning algorithms for collecting and classifying comments from a variety of online sources. The resultant data set was made available in the public domain to facilitate further investigation of this topic.

**Methods:**

We collected comments using Python-based web-scraping tools from the New York Times, YouTube, Twitter, and Reddit. We developed a set of guidelines for the creation of training data and manual classification of comments as either related to living organ donation or not. We then classified the remaining comments using deep learning.

**Results:**

A total of 203,219 unique comments were collected from the above sources. The deep neural network model had 84% accuracy in testing data. Further validation of predictions found an actual accuracy of 63%. The final database contained 11,027 comments classified as being related to living kidney donation.

**Conclusions:**

The current study lays the groundwork for more comprehensive analyses of perceptions, myths, and feelings about living kidney donation. Web-scraping and machine learning classifiers are effective methods to collect and examine opinions held by the general public on living kidney donation.

## Introduction

Kidney transplantation is the gold standard treatment for patients with end-stage renal disease (ESRD) [1] and can be much more cost-effective than dialysis [2]. Record numbers of transplants have taken place in recent years, but a shortage of donors persists despite recent increases [3]. Currently, the median wait time for a transplant is about 4 years in the United States, and close to 5000 patients die every year on the transplant wait list [4]. Living-donor kidney transplants generally provide better outcomes than deceased donor transplants but are inaccessible to many patients with ESRD, especially among certain racial and ethnic minorities [5,6], because of the potential burdens on donors. Such burdens can include financial costs related to donation and the risk of future kidney failure and death [7,8]. Over the last 2 decades, the US government has implemented programs that reimburse living donors for donation-related expenditures, such as the cost of traveling, medical costs for recovery and possible complications, and time away from the workplace. These programs are, however, known to have had little to no effect on the number of living kidney donors thus far [9].

Several studies have used qualitative approaches to identify possible barriers to kidney donation. These studies have identified several factors that can contribute to decision-making for both living and deceased donation, including the social influence of health care professionals (HCPs) [10], family members [11], and recipients and potential donors [12,13], as well as medical [14] and financial [15,16] barriers. Other factors are related to beliefs and concepts, such as unknown future needs [17] (ie, “What if my family member needs a donation someday?”), a desire for bodily integrity and choice, trust or mistrust of the health care system, religious and cultural beliefs, and a lack of information about donation [10]. Many of these studies, however, focus on identifying factors associated with deceased donation.

Additionally, the data have generally been derived from small samples of interviewees who have already participated in the donation process or from analyses of data from a single transplant center. As such, the extracted data are primarily representative only of those who have had direct experience in living donation. The viewpoints of the general public, who may be curious or have misconceptions about donation but have no direct experience in donation, are thus rarely represented. By leveraging the large volume of opinions and comments available online, this study represents a step toward better understanding of the public’s perception of living donation. At least one other research effort has taken advantage of comments on social media to investigate attitudes about organ donation. Jiang et al [18] found and analyzed 1507 reposts of 141 unique posts related to organ donation on the Chinese microblogging site Weibo; they were able to identify 5 major themes. The authors report that posts on “statistical descriptions” and the “meaningfulness” of organ donation prompted 3 and 2 users, respectively, to express the intention to become an organ donor. Henderson [19] performed a similar analysis.

The specific contribution of this study is the exploration of a machine learning classifier for the collection and analysis of a large database of labeled comments that were written by internet users and collected from multiple public sources. These comments reflect the users’ thoughts, feelings, and concerns regarding living kidney donation (LKD). The authors have made this database available upon request so that researchers on this topic can use the information for further analyses. The current study also examines and discusses the quality of predictions, highlighting particular areas of difficulty with regard to machine classification for further improvement.

## Methods

The comments were first collected and processed (the data processing phase). A small portion were then manually classified (annotated and labeled) for use as training data (the annotation phase). The training data were then used to develop a machine learning model that automated the classification process for large volumes of data (the modeling phase).

### Data Processing Overview

We created our data set through a process of gathering, filtering, and cleaning data [20]. Data were collected from different sources, including comments on newspaper articles published in the New York Times (NYT) and comments on the social media sites Twitter, YouTube, and Reddit. We manually annotated a small percentage of the data (1174 of 203,219 comments) and designed a neural network to classify the remaining comments. We separated the data set with 2 labels: related or unrelated to LKD. The characteristics of the training and testing data are shown in Table 1.

**Figure 1:**
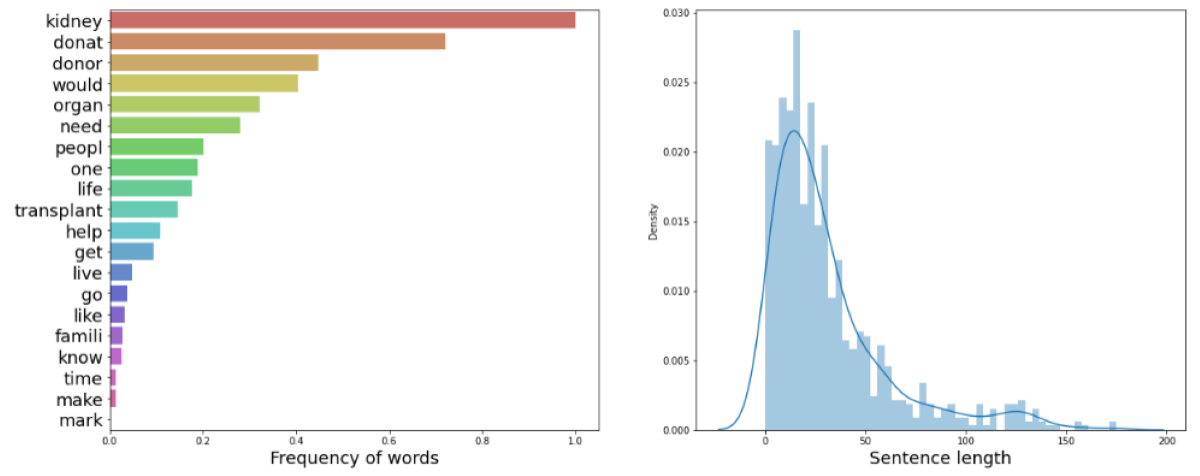
Word frequency and distribution for training data.

**Figure 2:**
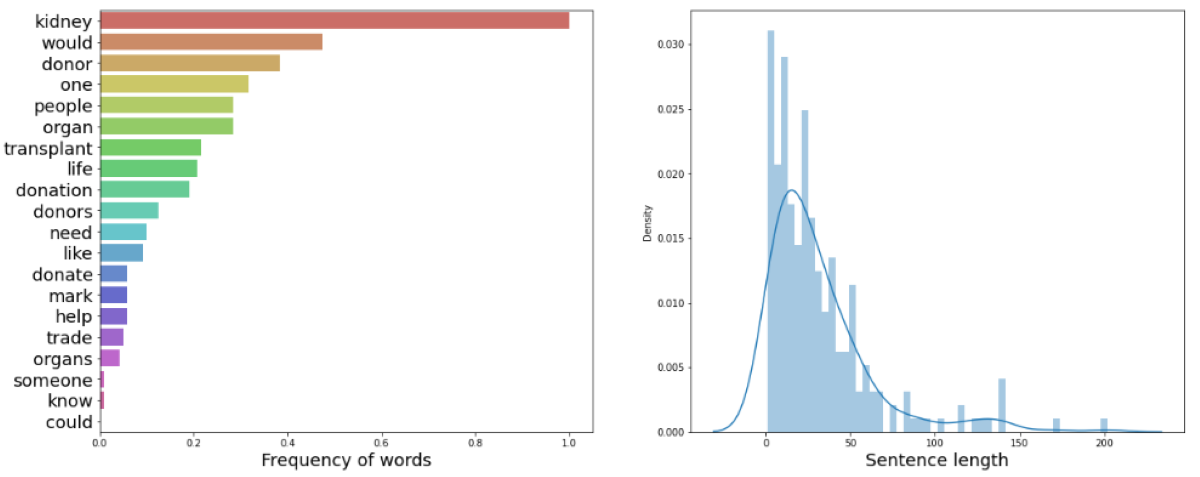
Word frequency and distribution for testing data.

Figures 1 and 2 illustrate the frequency and distribution of the words in the training and testing data, respectively. The training and testing data were compiled before all the comments were collected, so transfer learning was utilized for the final classification of the Reddit and YouTube comments [21]. The transfer learning model was validated to work sufficiently well on Reddit and YouTube comments by manually inspecting predictions.

**Table 1:** Characteristics of training and testing data.

Source	Training data (N=934)	Testing data (N=240)
New York Times comments, n (%)	312 (33.5)	83 (34.5)
Tweets, n (%)	622 (66.5)	157 (65.4)
Average words per comment, n	63.2	64.4
Maximum words per comment, n	380	381
Minimum words per comment, n	2	3

### Data Collection

To automate the process of downloading comments from the web, we used the Pushshift.io service for Reddit, Selenium for YouTube, and the application programming interfaces (APIs) from Twitter and the NYT. For each web source, we used search terms aimed at capturing content associated with LKD, while also excluding undesired content (such as political fundraising, which would otherwise appear in searches for terms like “donation”). More details on this exclusion process can be found in Multimedia Appendix 1. Tweets were captured via a live stream over time, but comments from the other 3 sources were captured from any date range allowed by the respective APIs. As YouTube had no API that suited our purposes, we manually searched YouTube for the term “living kidney donation” and identified 17 relevant videos with at least 30 comments each. Table 2 shows how many comments were collected, and over what time period, from each source.

**Table 2:** Summary of date ranges and numbers of comments (N=203,219).

Source	Date range	Unique comments, n
Twitter	Oct 2020-Apr 2021	148,662
Reddit	Jan 2010-Apr 2021	43,382
New York Times	Jan 2008-Apr 2021	6559
YouTube	Feb 2005-Apr 2021	4616

### Class Label Definition

The manual labeling of training data was one of the most important tasks in this study. The purpose of this classification labeling was to determine if a given comment was related to living organ donation. The annotation team worked through 1174 randomly selected comments and determined how each comment should be classified. We assumed at this stage that every comment from every source had equal weight. The process began with 3 annotators collaborating to classify a set of 403 comments, aiming to reach agreement on how the comments should be classified. The remaining 771 comments were classified after the decision criteria were more thoroughly established (the final criteria are described in the following section).

### Handling Ambiguity and Other Complexities

Annotations began with a simple idea: capture the comments that mention LKD. But the convoluted reality of human language is rarely simple enough for easy classification, and nuances abound. For example, can we assume a person’s sentiments on deceased donation carry over to their opinions on living donation? How should we classify comments in which people express their thoughts on a policy related to LKD even if they do not say whether they personally would donate? To overcome this obstacle, each annotator was given a set of classification criteria to determine whether a comment should be classified as “related.”

Even with the explicitly defined classification criteria, the annotation team still encountered significant difficulty in reaching a consensus on many of the comments. During the first stage of annotation, of 403 comments to be annotated, 124 were not classified unanimously. A few guiding principles emerged as the team discussed the dissenting comments. First, while comments explicitly mentioning organ sales and conversations about the illicit organ trade were excluded, the criteria were expanded to allow most other comments that involved cost or finance-related policies about organ donation.

**Figure 3:**
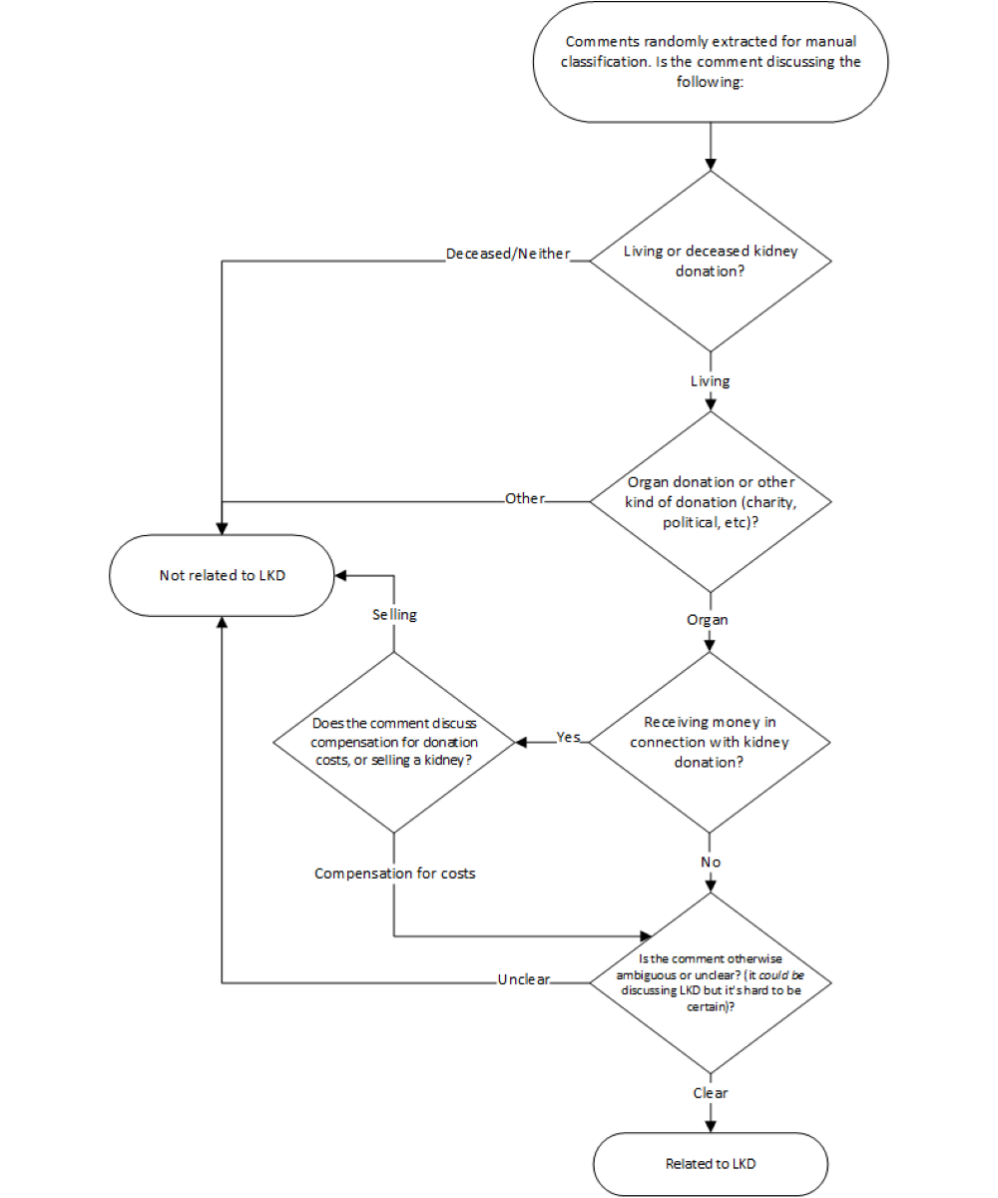
Classification criteria for manual labeling of training data. LKD: living kidney donation.

The second principle was to reverse an initial position about encouraging annotators to select “yes” in cases of uncertainty and ambiguity and to instead select “yes” only when they were confident doing so. This last criterion was to clarify that each comment must be viewed as independent from all the other data and that the human annotators should not consider the larger context (ie, other comments in the discussion) or make inferences. This last adjustment represents an important distinction in the way that humans learn compared to the way that machines learn. It is important to note that these criteria forced us to exclude data that could ultimately have been meaningful in order to obtain better performance for the overall model. A flowchart illustrating the decision criteria process is shown in Figure 3. We note that the comments determined to be “not related” were not quantified by the exclusion criteria during the annotation process, so numbers are not available.

### Modeling

**Figure 4:**
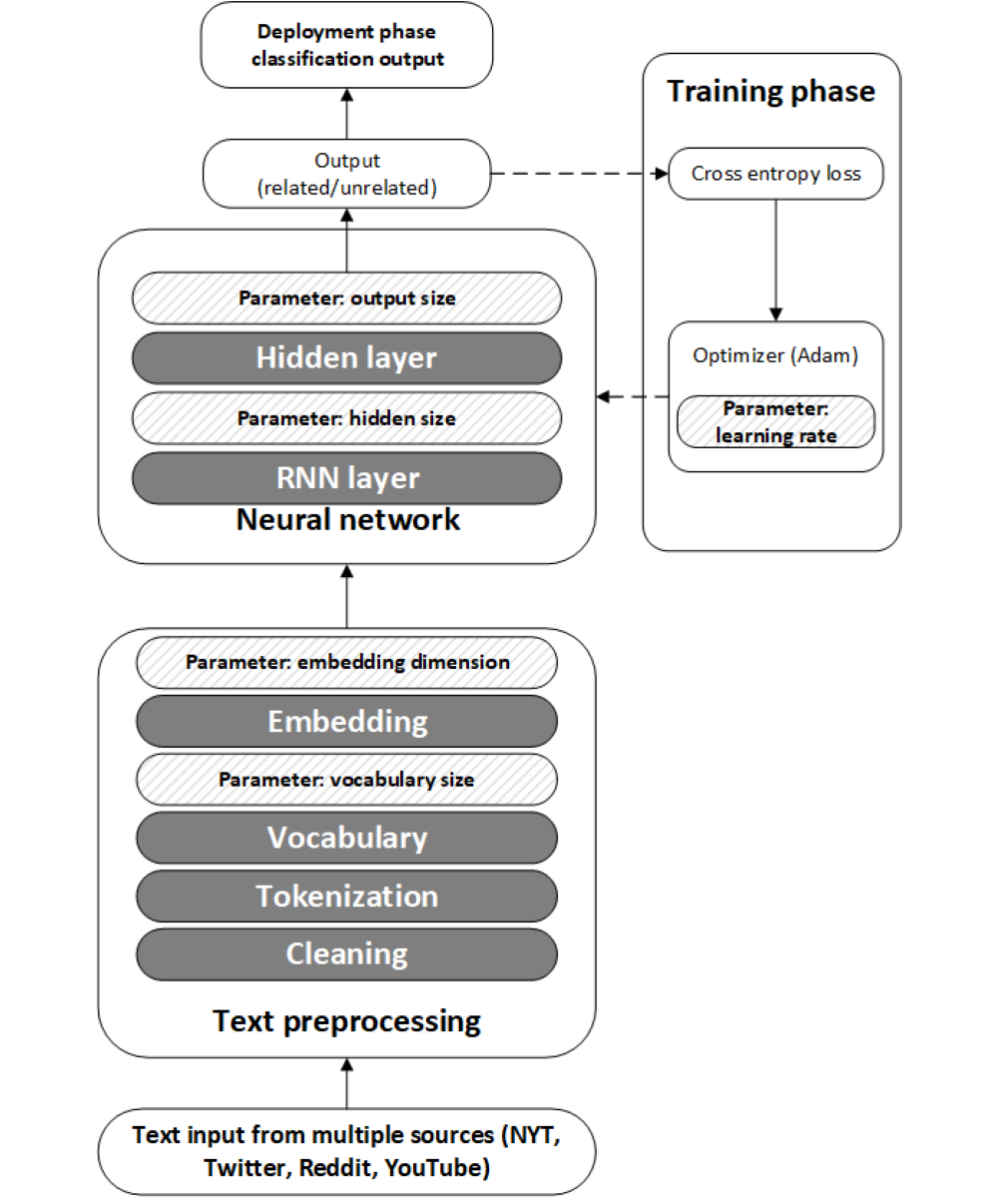
Neural network architecture. NYT: New York Times; RNN: recurrent neural network.

We developed a deep neural network to perform automated classification of the remaining comments (Multimedia Appendix 2) using PyTorch 1.11 in Python (version 3.8; Python Software Foundation). The network architecture is shown in Figure 4, with the hyperparameters illustrated by shaded boxes. Table 3 shows possible values for these parameters, each of which was evaluated to determine the best model.

**Table 3:** Neural network parameters and corresponding experimental values.

Parameter	Range
Tokenization level	Word, character
Embedding layer size	500, 600, 700, 1204, 2048
Hidden layer size	20, 30, 50, 100, 150, 200, 400, 500, 600
Learning rate	0.01, 0.001, 0.0001, 0.00001, 0.000001
Batch size	8, 16, 32, 64, 128

### Text Preprocessing

Prior to analyzing the text, documents were cleaned and normalized. The purpose of this text processing was to separate meaningful words from noise. This involved removing strange characters (eg, ¬ and ±), HTML tags, URLs, unnecessary repeated characters (“pleeeease” to “please”), number-character combinations (“401k”), adjusting contractions (“I’ve” to “I have”), and emojis. Words were also stemmed, so that words with the same root but different suffixes (such as “donate,” “donating,” and “donated”) would be treated as the same word (becoming “donat”).

**Figure 5:**
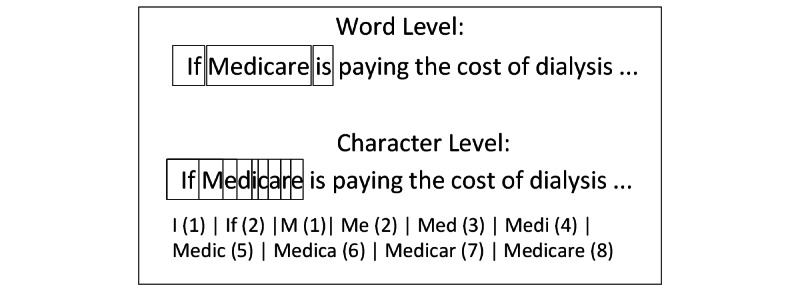
Illustration of word and character tokenization.

Tokenization was also performed at this stage. Tokenization is the process of separating sentences into smaller parts, such as words and characters. Word level tokenization is a split determined by a space between words, and character level tokenization is the process of dividing a word into different sections based on the length of characters. For example, we created 8 additional tokens from the word “Medicare,” as illustrated in Figure 5.

As the neural network cannot process text, we needed a layer to transform the vocabulary layer to numbers, a process called embedding. There are several techniques for this transformation, such as Google’s Word2Vec [22] and Stanford’s GloVe [23]. We experimented with these tools, but the specific domain of the text topics led to poor performance. To remedy this, we fed our vocabulary (as illustrated in Figure 5) to the Pytorch embedding tool [24], which allows users to train their own embedding layer.

We defined our neural network architecture with 2 layers: a hidden layer (where transformations take place) and an output layer (which determines the final classification). The hidden layer consisted of recurrent neural network nodes that were constructed with a long short-term memory cell [25]. We generated the probability for the output layer such that if the output layer generated a number greater than zero and less than 0.5 for a given comment, it was classified as not related; if it was between 0.5 and 1, it was classified as related. We used CrossEntropy [26] to define the loss function for the training process [27] and used the Adam optimizer [28] to optimize the neural network.

### Training and Evaluation Phase

**Figure 6:**
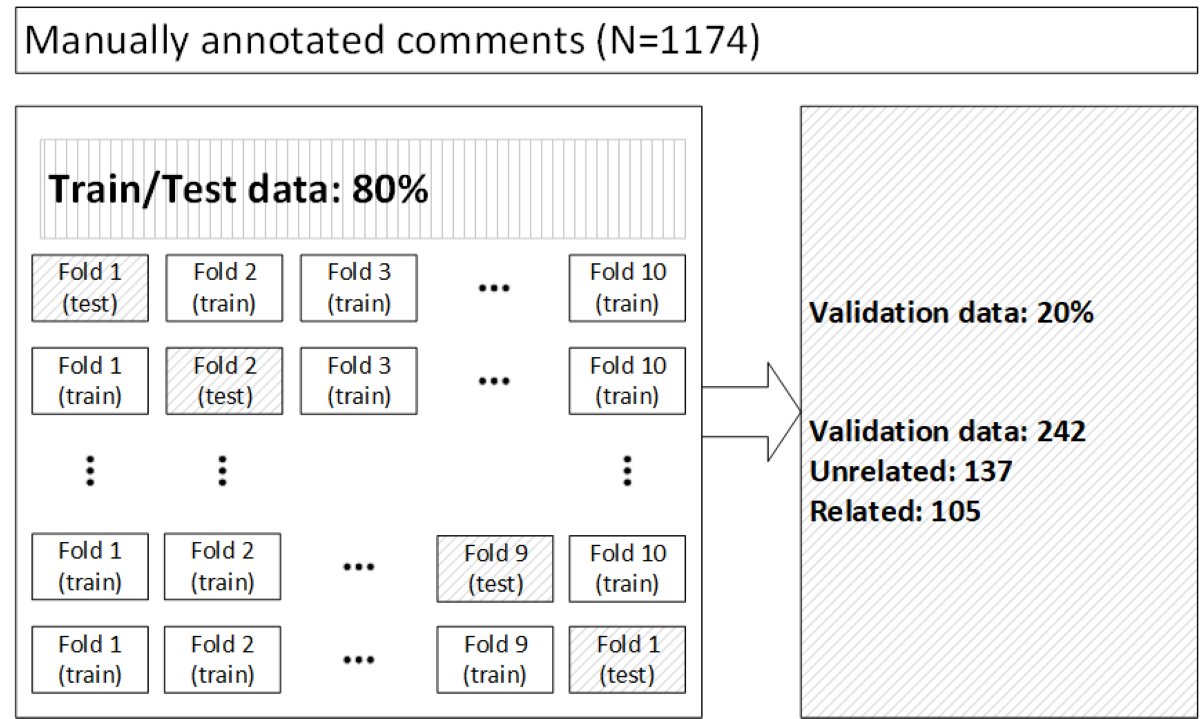
Structure of data training experimentation.

We used a nested K-fold validation procedure to guarantee the necessary model generality [29-31]. In the first iteration, we randomly separated 20% of the data to build the validation data set. The rest of the data (80%) were split into 10 separate folds to be iteratively used as training and testing data. Figure 6 shows the structure of the experiment. We selected K=10 so that we had 10 models to check against our test data set. The purpose of using K-fold cross-validation was to test how well the model could perform on unseen data by training it on small, separate chunks. K-fold validation was also considered useful in comparing the efficacy of word tokenization and character tokenization.

The metrics used to evaluate the performance of the classification model were precision (P), recall (R) and F1 score. The calculation of these metrics is explained in equations 1, 2, and 3, where related comments were treated as positive, and not related comments were treated as negative. The following notation was used: true positive (TP), true negative (TN), false positive (FP), and false negative (FN).



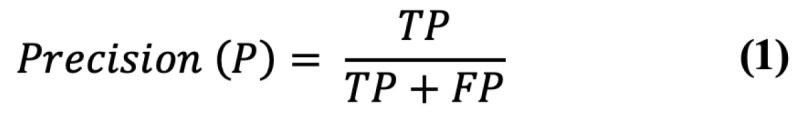





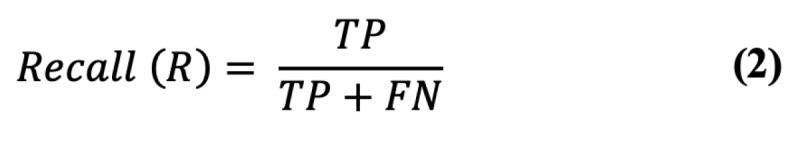





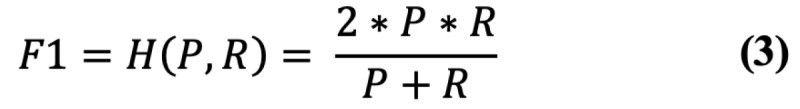



The precision metric measures how many related comments were correctly classified out of all comments that had been classified as related by the model. On the other hand, the recall metric indicates how many comments were correctly classified out of all the comments that were labeled as related by the annotation process. To select a winning model, the value of both precision and recall should be near 1. The F1 score is the harmonic mean of precision and recall; this measure provides a sense of model generalization. Accuracy (equation 4) is the number of correct classifications out of all classifications made.



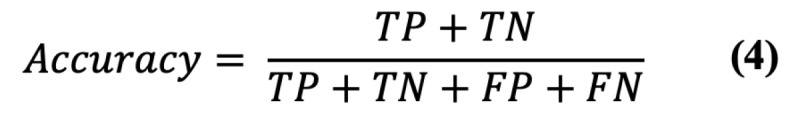



### Assessment of Machine-Classified Comments

After identifying satisfactory hyperparameters for the model, the model was used to automatically classify the complete data set. To verify the quality of the automated results, a random assortment of 912 comments (219 for the NYT, 222 for Reddit, 187 for Twitter, and 284 for YouTube) for each prediction outcome (ie, “related” and “unrelated”) were read and given an indicator to determine if the classification was correct according to the classification criteria described in the section “Handling Ambiguity and Other Complexities.” False positives (ie, comments incorrectly predicted to be related) were further labeled to identify the error made by the classifier using the categories described in Table 4.

**Table 4:** Description of false-positive error types.

Classifier error type	Description
Deceased donation	Comment was centered on deceased rather than living kidney donation
Figure of speech	Comment used phrases such as “I’d give a kidney” as a figure of speech or in a joking manner
Insufficient information	Comment had language that was too ambiguous to clearly determine its association with living kidney donation
Irrelevant	Comment was entirely unrelated to living kidney donation (see discussion section for more information)
Kidney stones	Comment mentioned kidney stones with no reference to living kidney donation
Non–living kidney donation policies	Comment expressed opinions on policies related to kidney donation, such as opt-out versus opt-in or legalization of kidney sales, with no information about how such policies might affect the commenter’s personal decision regarding donation
Recipient, dialysis, or kidney failure	Comment discussed challenges specifically for (or from the perspective of) a potential kidney recipient, such as kidney failure and dialysis; no information about living kidney donation
Selling or money	Comment discussed the monetary value of a kidney (specifically not used as a figure of speech or joke)

### Ethical Approval

The University of Louisville Institutional Review Board provided approval exemption for this study (22.0458).

## Results

In this section, we show the quantitative outcomes of our analysis. A testing accuracy of 84% was achieved using the following model hyperparameters: 10-character-gram tokenization, 700 embedding layers, a batch size of 8, 50 hidden layers, and a learning rate of 0.00001. Additionally, precision, recall, and F1 score each achieved 84% in the test data. Once the neural network was trained to achieve the above results, it was used to automatically classify the remaining comments. This yielded 11,027 related comments and 192,192 unrelated comments. Results from further evaluation of the predicted values, as discussed in the section “Assessment of Machine-Classified Comments,” are shown in Tables 5 and 6, sorted by comment source. Additional details on the further evaluation can be found in Table S7 in Multimedia Appendix 3.

Table 7 shows the distribution of false positive errors by social media source. We note that many of the irrelevant YouTube comments can be attributed to a single popular video showing an interview with a celebrity whose friend donated a kidney to her.

**Table 5:** Summary results for sensitivity and specificity of postclassification data.

Sources	False positives (N=576)	True positives (N=336)	False negatives (N=100)	True negatives (N=812)
New York Times, n (%)	107 (18.6)	112 (33.3)	19 (19)	200 (24.6)
Reddit, n (%)	146 (25.3)	76 (22.6)	27 (27)	195 (24)
Twitter, n (%)	159 (27.6)	28 (8.3)	7 (7)	180 (22.2)
YouTube, n (%)	164 (28.5)	120 (35.7)	47 (47)	237 (29.2)

**Table 6:** Summary results for F1 macro, precision, recall, and accuracy of postclassification data.

Sources	F1 macro (total score of comments was 60.2%), %	Precision (total score of comments was 36.8%), %	Recall (total score of comments was 77.1%), %	Accuracy (total score of comments was 62.9%), %
New York Times	70	51.1	85.5	60.7
Reddit	58	34.2	73.8	47.1
Twitter	46.8	46.8	15	46.8
YouTube	61.2	61.2	42.3	46.2

**Table 7:** Count of error types by source.

False positives	New York Times (N=107)	Reddit (N=146)	Twitter (N=159)	YouTube (N=164)	Total (N=576)
Deceased donation, n	16	10	0	10	27
Figure of speech, n	0	2	43	3	48
Insufficient information, n	9	39	6	15	69
Irrelevant, n	39	80	60	114	293
Kidney stones, n	0	0	15	0	15
Non–living kidney donation policies, n	25	4	0	2	31
Recipient, dialysis, or kidney failure, n	17	9	23	27	76
Selling or money, n	1	2	12	2	17

**Figure 7:**
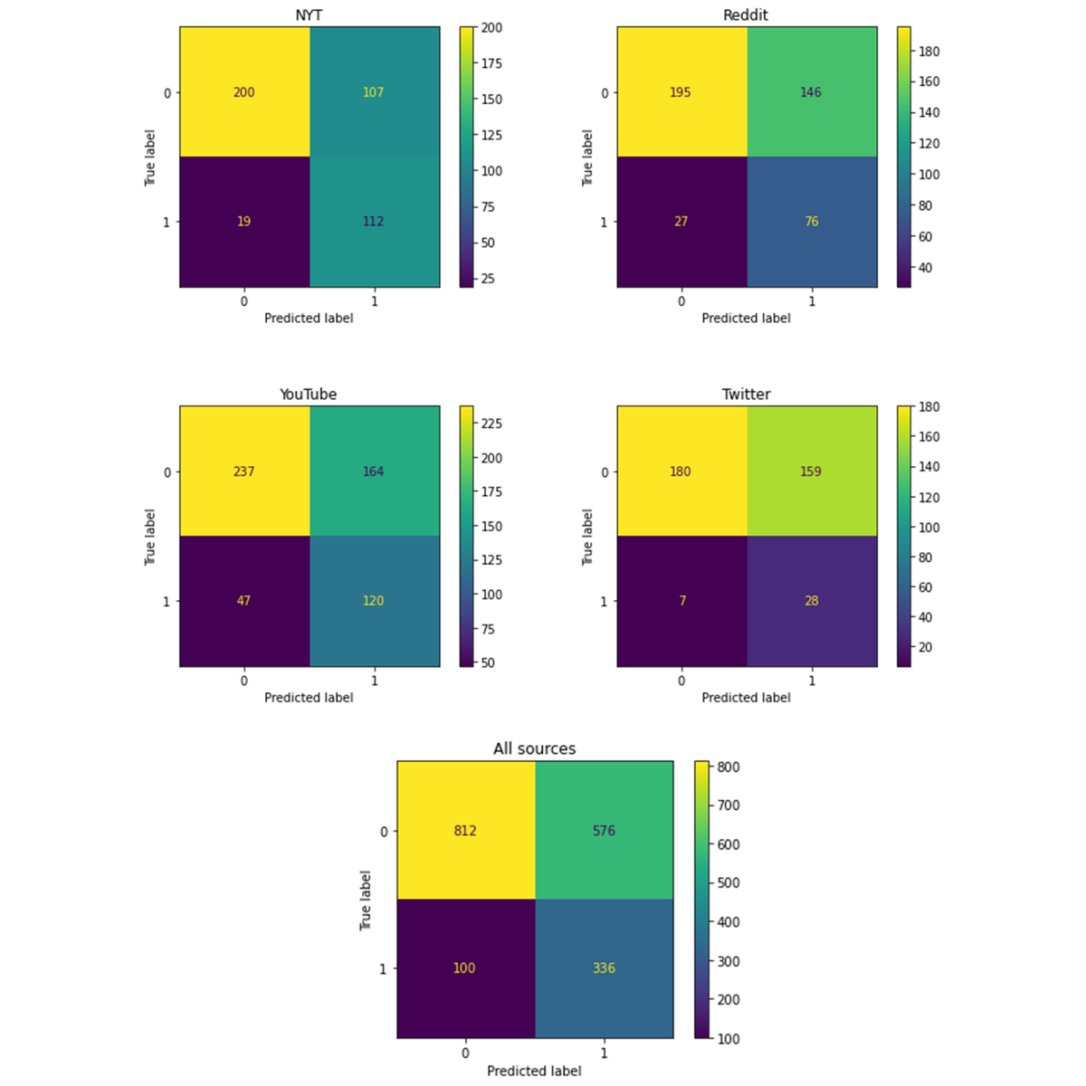
Confusion matrices for New York Times, Reddit, YouTube, Twitter, and aggregated comments. Clockwise from the top left corner, each quadrant of the confusion matrix shows the true negatives, false positives, true positives, and false negatives. An ideal model will produce quadrants in the top left and bottom right whose color is associated with high values (bright yellow colors), and quadrants in the top right and bottom left whose color is associated with low values (very dark purple colors). NYT: New York Times.

Figure 7 shows the confusion matrices for predictions made based on comments from the NYT, Reddit, Twitter, and YouTube, respectively, followed by the confusion matrix for the comments from all sources (in aggregate). We observe that for each source—and overall—the model had greater numbers of false positives than false negatives, illustrating a tendency to overpredict comments as being related.

We observed that 107 of 336 (32.3%) of comments in the related categories were on the topic of personal relationships (Table S7 in Multimedia Appendix 3), which can reasonably be expected, as these are currently the most common type of living donations that take place. We also observed that 293 of 576 (50.1%) of false positives (ie, comments incorrectly predicted to be related) were in the irrelevant category. This category produced the greatest number of false positives from each source. Table 8 shows the other top 2 categories that were most prevalent in misclassifications, along with an example comment to illustrate each.

**Table 8:** Top 2 categories that were most prevalent in their misclassifications, with example comments. Comments are shown “as-is“ after undergoing preprocessing.

Sources/categories		Example comments
**New York Times**		
	Non–living kidney donation policies (25/107 misclassified comments)	“how about making organ donations an opt out process instead of opt in everyone is automatically an organ donator unless they opt out several european countries do this with much success”
	Recipient, dialysis, or kidney failure (17/107 misclassified comments)	“my mom was on dialysis for years and died at the age of i was seeing what she went through i would never use dialysis i would get my affairs in order make my peace with god and simply fade away”
**Reddit**		
	Insufficient information (39/146 misclassified comments)	“it really sucks but at that age i wouldn’t even give my grandma one it probably wouldn’t even be recommended”
	Deceased donation (10/146 misclassified comments)	“the point is that when you re dead you re dead being on the donor list is the right thing to do no matter what and there is nothing that anyone can say to change that there is no excuse for not being a donor in my eyes”
**Twitter**		
	Figure of speech (43/159 misclassified comments)	“i m going to this even if i have to sell my kidney”
	Recipient, dialysis, or kidney failure (23/159 misclassified comments)	“when good things happen to good people my friend s husband finally got a kidney”
	Kidney stones (15/159 misclassified comments; this category was unique to Twitter)	"i don t know if it is a kidney stone all i know is it s been days and isn t letting up i thought i maybe pulled a muscle but this isn t muscle pain for sure"
**YouTube**		
	Recipient, dialysis, or kidney failure (27/164 misclassified comments)	“i ve been on dialysis for almost a year I am i m going next week for my evaluation the while process scares me so bad it s so hard but i want it so bad i ll do anything to be normal again”
	Insufficient information (15/164 misclassified comments)	“mad respect for this man i would like the courage to do something like this one day”

## Discussion

### Principal Findings

This study confirmed that the comments available from the internet can provide data on the general perception of living donation. Our trained model identified 11,027 comments related to LKD and 192,192 comments unrelated to LKD. Above, we present a sample distribution of comments that were incorrectly classified and their associated error types. There was a great deal of nuance and subtlety in the comments that could cause confusion for human classifiers, further increasing the difficulty for the machine classifier.

Many users wrote comments expressing their opinions regarding current policies. Though there was disagreement regarding how, nearly all users were supportive of making organs and transplants more accessible. There was notable support for a policy that would give preference or priority to designated or past organ donors when they face the need for organs. In the context of compensation for donation costs, it was also common to observe conversations regarding the legalization of organ sales. The two sides of this were primarily concerns about taking advantage of vulnerable populations and confidence in ethical market self-regulation. The various sources from which the comments were retrieved provided different kinds of comments. Comments that contained opinions about policy were most likely to be retrieved from the NYT, though they were also common on Reddit. There were also several self-reported accounts in the NYT and YouTube comments of someone or their spouse having previously been a living donor.

The character-restricted nature of Twitter meant that comprehensive ideas were less likely to be captured. Twitter was also more likely to produce comments in which people asked for donations or advocated for a loved one in need of an organ. Meaningful comments from YouTube were more often from people who had previous experience with transplants, either as patients or donors. While many of the Reddit comments were of little use, the “ask me anything” (AMA) subreddit provided a veritable treasure trove of information. There were threads written by people who had donated altruistically and invited people to “AMA.” This format, more than any other we encountered, seemed to yield the most thoughtful questions, concerns, and even resolutions to those concerns (to paraphrase one such person upon learning about a voucher system for the donor’s loved ones: “I’ve considered doing this before and never actually [done] anything. This has inspired me to sign up. Thank you!”).

Though there were positive responses from many users, some users were more cynical. One such user expressed the following: “the risk to living donors is also downplayed...people are guilted into acting as living donors only to find themselves at greater risk down the line.” Others wrote about frustrating experiences with the medical system or other worries, but we did not observe any blatantly false ideas in the comments. Lack of information was much more common than possession of misinformation.

To efficiently compile relevant information from comments and opinions found on the web, we used deep neural networks trained with specific criteria-driven classification labels. With this approach, we were able to develop a model that could identify comments related to LKD with an expected accuracy of 84%. Though further work remains to refine these results and classify these related comments according to the relevant factors, this first stage of classification indicates that the method could potentially be a valuable tool to extract themes related to barriers to and motivations for living donation. Because the topic is so nuanced, well-defined classification criteria for training data will be a vital part of developing a successful model. It is vital to have multiple people collaborating on training data annotation to ensure uniformity. Without these measures, the viability of this approach becomes less certain.

We note that the sizeable number of comments classified as irrelevant was to be expected to some extent. We suggest the following reasons to explain why our model incorrectly classified irrelevant comments as related to LKD: First, the size of the training data was relatively small compared to the total number of comments classified (1174/203,219). We project that with more (and more correctly labeled) training data, the model would yield better predictions. Second, models based on neural networks tend to have generalization errors that are sometimes identified as gaps [32]. Third, as mentioned above, there exists a great deal of nuance in this topic, and certain words that have no real significance may appear to the model as being important. For example, “parts” could be seen as a word that indicates “parts” of a body (ie, an organ), but it is simply a common word used in many settings.

For deceased kidney donation, there are a handful of studies that have utilized modern computer-science methods to analyze motivations and challenges associated with kidney donation. A recent study [33] discussed the use of natural language processing to glean information about deceased donors and the prospective utility of their kidneys. This information was retrieved from the United Network for Organ Sharing’s DonorNet program, in which organ procurement organizations enter raw text about the donors’ medical and social history, the history of their admissions, and other noteworthy information. A similar study [34] gathered 342 Spanish articles that contained the text “donacion de organos.” The authors found that a positive perception of kidney donation may be a contributing factor to the high rate of kidney donation in Spain. In another study [35], social media posts were collected to study the limitations of social messaging campaigns for deceased kidney donation.

Through the process of manual classification of training data, we observed nearly all barriers noted in the prior literature listed above, as well as early indicators of patterns. For example, the data suggested that the most frequent factors seen in the comments were directly related to the potential impact on prospective donors: considerations of immediate costs and risks of donating and the consequences of such a decision on those close to a donor. Broader influences, such as culture and belief systems, the influence of family members, and perceptions of the medical system, were less relevant to decisions related to living donation and more relevant to decisions related to deceased donation. In our manually labeled data, we did not observe the influence of HCPs as a factor that influenced a prospective donor’s decision to donate. Prior research indicates that barriers to donation attributable to HCPs include, for instance, lack of communication between transplant and dialysis teams, lack of training and information among HCPs, and negative attitudes held by some HCPs toward LKD [10].

Our study also recognized that the content and the quality of comments varied rather significantly depending on where they were retrieved. The AMAs of Reddit invited people to ask whatever questions they had, to be answered by someone who had been through the process personally. The downside of this particular resource is that there were only a few AMAs from living kidney donors. Comments from the NYT were more dependent on the content of the article to which they were attached, had no dialogue with the author, and were more conducive to debates on policy than to answering questions from curious prospective donors. Further analysis may provide greater insights into what kinds of internet sources yield the most meaningful information.

### Limitations and Future Work

These collected data provide several opportunities for research on LKD. The data can be used for more complicated analysis, such as topic modeling and clustering, with the purpose of detecting barriers and motivations in multisource data sets. Future work may consider the following: instead of a first-stage binary classification, it may be beneficial to consider 4 classifications, such as “irrelevant,” “recipient-related,” “deceased donation,” and “LKD-related.” As deceased donation and recipient-related issues are commonly intertwined with conversation about policies, such identification may also help mitigate the misclassification of those topics and reduce the number of entirely irrelevant comments that are erroneously classified as related. Other methods, such as multi-task learning models, could make predictions for comments based on their media source without requiring an independent model for each source.

Additionally, we assumed that each comment should be read independently to aid the model classification. However, it is sometimes possible to maintain an association between comments. For example, in Reddit, each comment has an ID, and if it is a reply, there is a parent ID connecting it to the original comment to which the user is replying. By using this association, the assumption of independence may not be necessary, because it can be better understood that the comment is being written (or not written) in the context of LKD. This would likely help reduce the number of comments which—alone—do not contain enough information to determine their relevance to LKD (“insufficient information”).

We observed that there was very little propagation of myths or blatantly false ideas. Among comments that discussed deceased donation (ie, that were unrelated to LKD), there were cynical comments that doctors might reduce life-saving efforts for a dying patient so that an organ could be harvested quickly. While cynicism or frustration with personal experiences appeared in some related comments, misconceptions about LKD were usually nested in expressions of fear or concern (the “risk of donation” category, for example). We suggest that users are more likely to have no (or very little) information about LKD than to have incorrect information. The comments generally indicated that people were curious and prone to ask questions about LKD and wanted to make suggestions about how to increase the number of living donors.

We also acknowledge that more comments could be added to the training data, as the given number of labeled comments was a result of the time-consuming nature of the annotation process. In this exploratory study, we focused on estimating the necessary sample size through a human-annotation process and defining possible labels for the first time. The labeled comments are available upon request from the authors. Finally, we acknowledge that this data is not necessarily representative of all populations. Though internet access continues to expand globally, the distribution of users is not uniform, and each source will have different user bases. For example, according to the 2022 Global Digital Overview Report [36], Reddit users are twice as likely to be men than women, and other studies, discussed in Amaya et al [37], have estimated that between 80% and 90% of global Reddit users are aged 18 to 34 years. Each other source is likely to have its own unique demographic features that should be considered when making inferences from the data.

There is a significant need to understand why people do or do not choose to be living kidney donors. Although prior literature has made contributions toward understanding the context surrounding donation, there is no publicly available data set with information about the thoughts of the broader population on the matter. This project has taken one step toward filling this gap by scraping 203,219 unique internet user comments and tweets and developing a machine-learning classification model to identify comments related to LKD. The documents classified as relevant to LKD were compiled into a single database and are available upon request from the authors. With this database, the groundwork has been laid for more comprehensive analysis of the feelings and ideas that people have surrounding LKD. The data could also be used to identify common misconceptions about donation or information that could lead to changing minds. While rigorous classification of decision-making factors remains to be performed, the findings from this study show that machine learning is a promising tool for the capture and classification of internet comments related to LKD.

## Appendix

Multimedia Appendix 1 Additional details on data collection process.

Multimedia Appendix 2 Additional information regarding the use of neural network classifiers.

Multimedia Appendix 3 Additional information regarding the evaluation of predicted data.

## Abbreviations

AI: artificial intelligence
AMA: ask me anything
API: application programming interface
ESRD: end-stage renal disease
FN: false negative
FP: false positive
HCP: health care professional
LKD: living kidney donation
NYT: New York Times
P: precision
R: recall
TN: true negative
TP: true positive
