# Supplementary material for: Classifying Comments on Social Media Related to Living Kidney Donation: Machine Learning Training and Validation Study
Source: JMIR Med Inform. 2022 Nov 8;10(11):e37884. doi: 10.2196/37884 (PMC9682456; doi:10.2196/37884)
Supplement: Multimedia Appendix 1 [file medinform_v10i11e37884_app1.docx]

JMIR LKD Analysis Appendix A

The search terms we used for selecting Twitter comments related to living organ donation are as follows: [“save kidney”, “save a life kidney”, “life saving kidney”, “life kidney”, “kidney”, “dialysis kidney”, “donated kidney”, “donate kidney”, “OrganDonation kidney”, “Organ donation kidney”, “kidneyfailure kidney”, “transplant kidney”, “kidneytransplant kidney”, “DonateLife kidney”, “TransplantTwitter kidney”, “kidneydisease kidney”, “organ donor kidney”, “surgery kidney”, “-visa card” , “-funds”, “-raising money”, “-gofundme”, “-collect”, “-anything helps”, “-contribute”] Where the minus sign (-) was used to exclude terms from the search. We paired every desired term such as “life” and “donate” with the word “kidney” to avoid results where people were asking for donations related to other things.

The NYT API searched for articles whose titles or stories contained any combination of the terms [“organ”, “kidney”, “donation”] between the start of 2008 and the start of 2021. The search resulted in 169 article links, but manual inspection of the articles found that only 16 contained comments related to LKD. This void of data can be partially attributed to the fact that, for many of the articles, there simply were no comments at all. For the remainder, LKD was not the topic the users discussed.

Pushshift.io was used to scrape any Reddit comments between the January 1, 2010 and April 1, 2021 containing the search terms [“kidney donor”, “kidney transplant”, “kidney donated”, “kidney donate”, “kidney years ago”, “kidney need”, “kidney stranger”, “kidney willing donate”]. As YouTube has no API, we manually searched the YouTube website using the search terms “living kidney donation” and identifying videos with at least 30 comments, whose links would be used to scrape with Selenium (a web-scraping Python library). Seventeen videos were selected, and 4,616 comments were collected.
