# Supplementary material for: Classifying Comments on Social Media Related to Living Kidney Donation: Machine Learning Training and Validation Study
Source: JMIR Med Inform. 2022 Nov 8;10(11):e37884. doi: 10.2196/37884 (PMC9682456; doi:10.2196/37884)
Supplement: Multimedia Appendix 2 [file medinform_v10i11e37884_app2.docx]

JMIR LKD Analysis Appendix B

**Deep Neural Network Classifier**

Neural networks are relatively novel classification methods - at least in terms of widespread use if not in concept - and are inspired by the human brain which is able to quickly identify, recognize, and categorize sensory inputs. It is common to see them used for classification problems involving images, but they have strong applicability in text analysis as well [37]. The fundamental concept in a neural network is that by processing input features through multiple layers containing multiple neurons, the entire system of neurons will be able to collectively identify important features of the input data and correctly make predictions on new data accordingly [38]. Each neuron in a layer receives an input from a previous layer, multiplies that value by a certain weight and outputs the new value to the next layer. By iteratively adjusting these weights over time, the model will “learn” which weights yield the best results.

**Long Short-Term Memory**

Long Short-Term Memory (LSTM) is a method that is often used when dealing with text data, rather than numerical. The key feature of an LSTM layer is that it can control whether and when to forget or retain the context of a given neuron (i.e., the input that was given to a previous neuron) [38]. Consider, for example, the following text: “John went to the store to get some eggs. He came back later with butter instead.” In this example, the word “He” refers to “John” and there is a relationship between “eggs” and “butter”. By using LSTM, the model learns how and when to retain these pieces of information, rather than viewing each word as independent features. Unlike traditional feed-forward neural networks, LSTM has a feedback connection. This characteristic helps process a sequence and connect the words in the learning process.

**Embedding Layer**

For some problems, such as online learning, the need to create a dictionary before training can be a nuisance. This is often solved with feature hashing, where a hash function is used to assign each token *T* to one of a fixed set of “buckets” 1, 2, . . . *B*, each of which has its own embedding vector [39]. A hash function is a function which maps data of arbitrary size to fixed-size values. For example, in a set of three documents containing 100, 200, and 500 words, the hash function will map the data from these documents into strings of 50 characters each, corresponding to the appropriate feature or “bucket”.

Because the objective of hashing is to reduce the dimensionality of the token space *T*, it is common to have a number of buckets that is significantly smaller than the number of unique tokens *T*. This inevitably results in many tokens “colliding” with each other because they are assigned to the same bucket (an event which is more likely to occur with the 500-word document, for example). When multiple tokens collide, they will get the same vector representation which prevents the model from distinguishing between the tokens. A hash embedding may be seen as an interpolation between a standard word embedding and a word embedding created using a random hash function (the hashing trick).
