# Supplementary material for: Classifying Comments on Social Media Related to Living Kidney Donation: Machine Learning Training and Validation Study
Source: JMIR Med Inform. 2022 Nov 8;10(11):e37884. doi: 10.2196/37884 (PMC9682456; doi:10.2196/37884)
Supplement: Multimedia Appendix 3 [file medinform_v10i11e37884_app3.docx]

JMIR LKD Analysis Appendix C

**Evaluation of predicted data**

Related comments (positives) were categorized by their content, described as follows. Several of these topics were first identified in the literature discussed in Section 1.

- Altruism – The comment discusses LKD by strangers or speaks of the goodness and generosity of someone who donates a kidney, without clear reference to an act done by someone with a personal relationship. Some overlap with “Paired donation chains” category.
- Bodily integrity – Expressing a desire to maintain the “wholeness” of one’s body, with phrases such as “we have two kidneys for a reason.”
- Compensation for donation costs – Discussion of the costs incurred by living donors.
- Future need – Concerns about “what if”; i.e., a kidney is needed later in life after making the choice to donate? This has strong overlap with the “Risk of donation” category
- Mistrust of “system” – Cynicism about the medical or governmental system, that it does not have the best interest of the patient at heart.
- Paired donation chains – Discussion about paired kidney exchanges. Has considerable overlap with the “Altruism” category
- Policies – Discussion about policies related to LKD. Some overlap with “Compensation” category.
- Providing information – A user is attempting to educate or explain to another user about how the kidney donation process works.
- Relational ties/Friendship – The influence of a personal relationship on the decision to donate.
- Reservation or inability (broadly) – An unspecified hesitation, refusal, or declaration of inability regarding LKD.
- Risk of donation/Medical concerns – Discussion about dangers and risks associated with LKD, as well as expressions of concern regarding the medical implications of donating. Some overlap with “Future need” category.
- Uncertainty/Lack of information – Asking questions about the donation process, acknowledging ignorance on the topic, etc.
- LKD broadly – Discussing LKD, but so generally that there is no indication of a more specific sub-topic.

Table 7 shows a breakdown of the evaluation performed on predicted data.

| **Evaluated Category** | **NYT** | **Reddit** | **Twitter** | **YouTube** | **Grand Total** |
| --- | --- | --- | --- | --- | --- |
| **INCORRECTLY predicted as “Related” (False Positives)** | **107** | **146** | **159** | **164** | **576** |
| Deceased donation | 16 | 10 |  | 1 | 27 |
| Figure of speech | | 2 | 43 | 3 | 48 |
| Insufficient Information | 9 | 39 | 6 | 15 | 69 |
| Irrelevant | 39 | 80 | 60 | 114 | 293 |
| Kidney stones |  |  | 15 |  | 15 |
| Non-LKD policies | 25 | 4 |  | 2 | 31 |
| Recipient/Dialysis/Kidney Failure | 17 | 9 | 23 | 27 | 76 |
| Selling/Money | 1 | 2 | 12 | 2 | 17 |
| **CORRECTLY predicted as “Related” (True Positives)** | **112** | **76** | **28** | **120** | **336** |
| Altruism | 14 | 5 | 5 | 18 | 42 |
| Bodily integrity | 3 | 1 | 1 | 1 | 6 |
| Compensation for donation costs | 21 | 5 |  | 7 | 33 |
| Future need | 6 | 3 |  | 4 | 13 |
| Mistrust of "system" | 5 |  |  |  | 5 |
| Paired donation chains | 8 | 4 |  | 3 | 15 |
| Policies | 2 |  |  |  | 2 |
| Providing information | 8 | 8 | 1 | 3 | 20 |
| Relational Ties/Friendship | 22 | 25 | 9 | 51 | 107 |
| Reservation or inability(broadly) | 2 | 3 |  | 8 | 13 |
| Risk of donation/ Medical concerns | 13 | 17 | 2 | 9 | 41 |
| Uncertainty/lack of information | 4 | 1 | 2 | 8 | 15 |
| LKD broadly | 4 | 4 | 8 | 8 | 24 |
| **Grand Total of “Related” predictions** | **219** | **222** | **187** | **284** | **912** |
| **INCORRECTLY predicted as “Unrelated” (False Negative)** | **19** | **27** | **7** | **47** | **100** |
| Altruism | 2 | 4 | 1 | 5 | 12 |
| Bodily integrity |  |  |  | 2 | 2 |
| Compensation for donation costs | 3 |  |  | 1 | 4 |
| Future need |  | 1 |  |  | 1 |
| Mistrust of "system" | | 1 |  |  | 1 |
| Paired donation chains | 1 | 2 |  | 1 | 4 |
| Policies | 3 | 1 |  |  | 4 |
| Providing information | 1 | 4 |  | 2 | 7 |
| Relational Ties/Friendship | 4 | 8 | 3 | 19 | 34 |
| Reservation or inability(broadly) | | | | 1 | 1 |
| Risk of donation/ Medical concerns | 2 | 2 | 1 | 3 | 8 |
| Uncertainty/lack of information | | 2 |  | 4 | 6 |
| LKD broadly | 3 | 2 | 2 | 9 | 16 |
| **CORRECTLY predicted as “Related” (True Negatives)** | **200** | **195** | **180** | **237** | **812** |
| **Grand Total of “Unrelated” predictions** | **219** | **222** | **187** | **284** | **912** |

Table 7 - Count of categories identified in post-classification comments
